# The Predictive Utility of Past Success: Skill and Chance in Children's Theory of Performance

**DOI:** 10.1111/desc.70123

**Published:** 2025-12-31

**Authors:** Hailey Pawsey, Jordan Bauman, Ayshe Ozlu, Stephanie Denison, Ori Friedman

**Affiliations:** ^1^ Department of Psychology University of Waterloo Waterloo Ontario Canada

**Keywords:** ability, chance, skill, theory of performance

## Abstract

**ABSTRACT:**

Success at a skill‐based activity shows that a person is competent and likely to succeed again in the future. Success at a pure‐chance activity, by contrast, does not imply competence or future success. In two experiments, we investigated children's developing understanding of how skill‐ and chance‐based activities differ in relation to competence. In both experiments, children aged 4–7 (total *N* = 279) saw skill‐ and chance‐based activities and judged whether a person who had previously succeeded with each activity would succeed when next attempting it. From Age 5, children were more likely to see past success as predictive of future success for skill‐ than chance‐based activities. The second experiment also looked at judgments about agents who had previously failed and found that children at all ages predicted future success similarly regardless of whether activities involved skill or chance alone. This experiment also included a sample of adults (*N* = 202), and found their responses were overall comparable to those of 7‐year‐olds. Together, these finding are informative about development in children's reasoning about the predictive utility of past success, and potentially about their *theory of performance—*their understanding of factors that determine whether agents are likely to succeed. The findings provide preliminary evidence for development in this theory at Age 5 while also showing that its development is protracted.

**Summary:**

- We examined how 4–7‐year‐olds distinguish between chance‐ and skill‐based activities.
- From Age 5, children saw past success as more predictive of future success for skill‐based activities.
- Children at all ages saw past failure as similarly predictive for both types of activities.
- Our findings suggest change in children's theory of performance at Age 5, while also revealing further improvements to Age 7.

## 1 Introduction

We investigate how young children distinguish activities that depend on skill from activities which involve chance alone. The distinction between these two kinds of activities is basic and important. It matters for both causal inference and for predicting future outcomes. Suppose Alice successfully performs a handstand twice in a row. Skill and ability are candidate causes for explaining her success (though luck could matter too), and her repeated success suggests that she will succeed again on her next attempt at a handstand. But if Alice rolls six with a die twice in a row, then skill and ability are unlikely to explain her success and her repeated success gives us no reason to expect that her next roll will also be a six.

The distinction between skill and chance may be straightforward for adults, but we were unsure about how or when children would recognize it. Questioning this might be surprising since much work in developmental psychology has looked at children's understanding of skill and ability (e.g., Folmer et al. 2008; Heyman et al. 2003; Nicholls 1978) and at their understanding of random processes where skill does not contribute (Denison et al. 2013; Goldberg 1966; Téglás et al. 2007). But, as we will review, most experiments looked at each kind of activity in isolation rather than in relation to one another, and it remains unknown how children come to distinguish between them.^1^

Investigating how children distinguish between skill and chance will be informative about children's *theory of performance*—their understanding of the factors that determine how individuals perform well and whether they are likely to succeed (Muradoglu and Cimpian 2020; Muradoglu et al. 2025). This theory can be used to understand and explain past successes and failures, and to predict whether future attempts will succeed. In particular, investigating how children differentiate skill from chance will be informative about how they view the predictive utility of past success for future attempts.

Looking at children's understanding of the distinction between chance and skill also advances the longstanding project in developmental psychology of capturing how young children understand people's actions. Early work focused on children's theory of mind—their understanding of how people's actions are affected by psychological states, such as beliefs, intentions, and desires, and also emotions, traits, and dispositions (for reviews, see Doan et al. 2025; Heyman 2009; Rakoczy 2022). Later work showed that children also recognize ways that actions depend on the external environment and the constraints and costs it imposes (Gergely and Csibra 2003; Jara‐Ettinger et al. 2016; Kushnir 2018). Distinguishing between skill and chance brings these competences together: Skill is specific to the individual and depends (in part) on internal psychological factors, whereas with pure chance the odds of success are determined entirely by the environment.

### 1.1 Skill‐Based Activities

Young children recognize that people differ in skill and ability and that these often contribute to success. For example, 3–5‐year‐olds judge that a story character who easily completes a puzzle is smarter (i.e., has greater ability) than another agent who completes the puzzle with difficulty (Heyman et al. 2003), and 4–5‐year‐olds recognize that a person who builds a block tower more quickly than someone else is better at building (Leonard et al. 2019; also see Gweon et al. 2017). Children's understanding of skill is also suggested by their judgments about how to best assign easy versus difficult activities, and how to allocate help. They understand that difficult activities should be assigned to those with more skill (Baer and Odic 2021; Magid et al. 2018), and that helpers should be assigned to agents completing difficult activities rather than easy ones (Bennett‐Pierre et al. 2018; Magid et al. 2018; also see Sierksma and Shutts 2020). In one study, 3–5‐year‐olds first played a hard game and an easy game and were later asked to assign one game to themselves and the other to a partner, who was either described as older or younger than themselves (Magid et al. 2018). Children mostly assigned the harder game to older partners and the easier game to younger ones.

Young children initially struggle to think about some aspects of skill and ability though. One difficulty is that younger preschoolers often equate greater skill with successful outcomes even when this is unwarranted. For example, many 3‐ and 4‐year‐olds judge that a slow runner who wins a race is a better runner than a faster one who lost because he tripped on a stone (Yang and Frye 2016). It is only at Age 5, that children robustly see the fastest runner as best. Similarly, although 4–5‐year‐olds are often successful in recognizing which of two structures will be more difficult to build from blocks, they struggle when the final structures are identical but differences in the starting states meant that one was more difficult to build (Gweon et al. 2017).

### 1.2 Chance‐Based Activities

Young children also appear to understand how chance and randomness determine outcomes when agents have no control—in situations where skill cannot contribute to success. For example, they understand that common options are more likely to be randomly selected than uncommon ones (Acredolo et al. 1989; Denison et al. 2006, 2013; Goldberg 1966; Yost et al. 1962).^2^ In one study, 3‐ and 5‐year‐olds saw a ball bouncing in a square that had three holes on one side and only one on the other (Téglás et al. 2007). The square was then occluded and children were told to press a key when the ball left the square (and was no longer occluded). Children at both ages responded more slowly when the ball came out the side with only one hole compared to the side with three holes, suggesting that they recognized it was more likely to randomly exit from the side with more holes. Children also appear to understand random processes when *evaluating* random outcomes (Doan et al. 2018, 2021; for a review see Doan et al. 2023). For example, from Age 4, they recognize that receiving two good and two bad candies from a machine with a low proportion of good candy is better than receiving the same candies from a machine with a high proportion of good candy, and from Age 5, they recognize that this will also make an agent happier (Doan et al. 2020).

Nonetheless, young children could think that skill bears on purely random processes. One hint comes from children's wishful thinking: Children often predict that unlikely but desired outcomes will happen in situations where they have no control (e.g., Bernard et al. 2016; Marks 1951; Weisz 1981; Wente et al. 2019). In one experiment, 3–6‐year‐olds were faced with six plastic eggs, which each concealed a winning token or a losing one (Hennefield and Markson 2022). Either children or the experimenter then chose one of the eggs and children were asked to predict what kind of token it contained. At all ages, children were more likely to guess the egg contained a winning token if they had chosen it. This difference could suggest that children thought success was not merely a matter of chance and that they impacted the outcome.

### 1.3 Comparing Skill and Chance

Only a few studies have directly compared children's judgments across situations where success depended on skill versus pure chance. Some early studies suggested that children under Age 8 do not differentiate between these two kinds of activities and think both are similarly affected by skill (Weisz et al. 1982; Thorkildsen and White‐McNulty 2002). For example, children in one study were asked to explain how players had succeeded at a game where the goal was to pick a card from an array of similar‐looking cards that exactly matched a target picture (Nicholls and Miller 1985). Children said success on the game depended on factors like age, intelligence, and practice. But they responded this way both for a skill‐based version where players could see the cards in the array, and for a chance‐based version where the cards were face‐down and players had to resort to pure guessing.

More recent work, by contrast, shows that young children readily recognize that agents guided by perception and knowledge are more likely to succeed than agents who lack it and must resort to guessing (e.g., Sodian et al. 2006). In one study, 3–4‐year‐olds were introduced to one puppet who knew exactly how a machine worked and another puppet who had never seen it before (Kushnir et al. 2008). When the machine activated after both puppets simultaneously put blocks on it, children thought the block placed by the knowledgeable puppet was the one that made it work. But if the puppets were blindfolded when they placed the blocks, children chose between them at chance. Success on such tasks, though, might not reflect a general understanding of the distinction between skill‐ and chance‐based activities. Children's success could instead depend on theory of mind and the specific understanding that perception and knowledge contribute to success, and possibly the belief that ignorance leads to failure (e.g., Chen et al. 2015; Fabricius et al. 2021; Ruffman 1996; but see Friedman and Petrashek 2009). If so, children might *not* differentiate skill‐based activities like balancing on a beam from chance‐based activities like rolling a die to a desired number—activities where the difference between skill and chance does not come down to the difference between knowledge and ignorance.

## 2 The Current Experiments

Our investigation centers on a key difference between skill‐ and chance‐based activities: Past success is more predictive of future success with skill‐ than chance‐based activities. With skill‐based activities, success depends on competence and past success provides evidence the agent has it. If someone whistles a song once, there is good reason to think they will be able to whistle it the next time they try—their success suggests they have the ability and skill needed to whistle it. With chance‐based activities, by contrast, past successes can only reveal the odds of success inherent in the task. If an agent flicks a spinner onto a desired color, this does not imply that they will succeed on their next try; success depends only on the odds offered by the spinner. Hence, children might recognize that past success is more predictive of future success for skill‐than chance‐based activities. But they could also be led astray by low‐level heuristics, for example, assuming past success always augurs success in the future.

We conducted two experiments on children aged 4–7. In both experiments, we told children about agents who had succeeded with a skill‐ or chance‐based activity and then asked children to predict how the agent would perform when attempting it again. In the second experiment, we also asked children about agents who were previously unsuccessful; in this experiment, we also included a sample of adults to get a sense of development of these judgments beyond Age 7. In both experiments, we asked children about a variety of familiar activities. For instance, skill‐based activities included whistling a tune and jumping as high as a desk, and chance‐based activities included winning at rock paper scissors and flicking a spinner to land on yellow.^3^ One potential disadvantage of our approach is that the skill‐ and chance‐based activities were quite different from one another—for instance, they were not matched like the skill‐ and chance‐based versions of the card game used in Nicholls and Miller (1985). Nonetheless, we chose this approach for two reasons: (1) We thought that asking children about familiar activities would be more likely to reveal competence compared with asking them about unfamiliar ones. (2) With the alternative approach of using skill‐ and chance‐based versions of the same task, it is difficult to isolate the effect of skill‐versus‐chance. For instance, in the card game, success was far likelier in the skill‐based version where agents could see the cards than in the luck‐based version where they could not, a difference that could strongly affect predictions of future success. Asking children about a variety of activities of each type reduces the chances of other differences between the activities driving the overall findings.

## 3 General Methods

Data and code are available at https://osf.io/kup2b/. Children were mostly tested at schools and daycares in the [region anonymized] (99% in Experiment 1 and 95% in Experiment 2); the rest were tested online via Zoom. Demographic information was not collected from each child (as per allowances of our IRB application). However, 64% of residents in the region are White; South Asians are the largest visible minority; these figures were supported by a census of students in [region anonymized] School Board, where most children were tested. In Experiment 2 we also tested adults in the United States recruited using Prolific.

Children completed the experiments in testing sessions where they also completed other experiments. Testing sessions took less than 15 min, and the experiments were on different topics and used different stimuli, scripts, and test questions.

We analyzed the results using generalized estimating equation (GEE) models run in R using *geepack* (Højsgaard et al. 2006) and used *emmeans* (Lenth 2023) to produce omnibus analyses and to run post hoc comparisons. We used *ggeffects* (Lüdecke 2018) to plot the models and to examine 95% confidence intervals (CI). Examining these intervals provided conservative estimates of when responses first exceeded or fell below chance, and when responses first diverged across conditions.

## 4 Experiment 1

### 4.1 Methods

#### 4.1.1 Participants

We tested 119 4–7‐year‐olds (*M*_age_ = 6;0, range = 4;0–7;10, 66 girls and 53 boys). Two other children were tested but excluded. One only responded to half the test trials, and the other child responded to none. In this experiment, we aimed to test 30 participants per age in years, but the actual numbers slightly departed from this.

#### 4.1.2 Materials and Procedure

**FIGURE 1:**
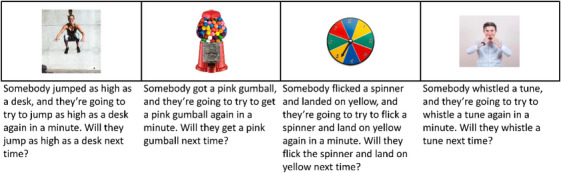
Experiment 1: Slides and testing script. Children completed the trials in a fixed order as shown here.

Children completed four trials, with two showing skill‐based activities and two showing chance‐based activities; see Figure 1. In each trial, children were told that someone completed the activity and would try to complete it again in a minute. They were then asked if the person would succeed again. The experimenter did not provide feedback on children's responses.

### 4.2 Results

**FIGURE 2:**
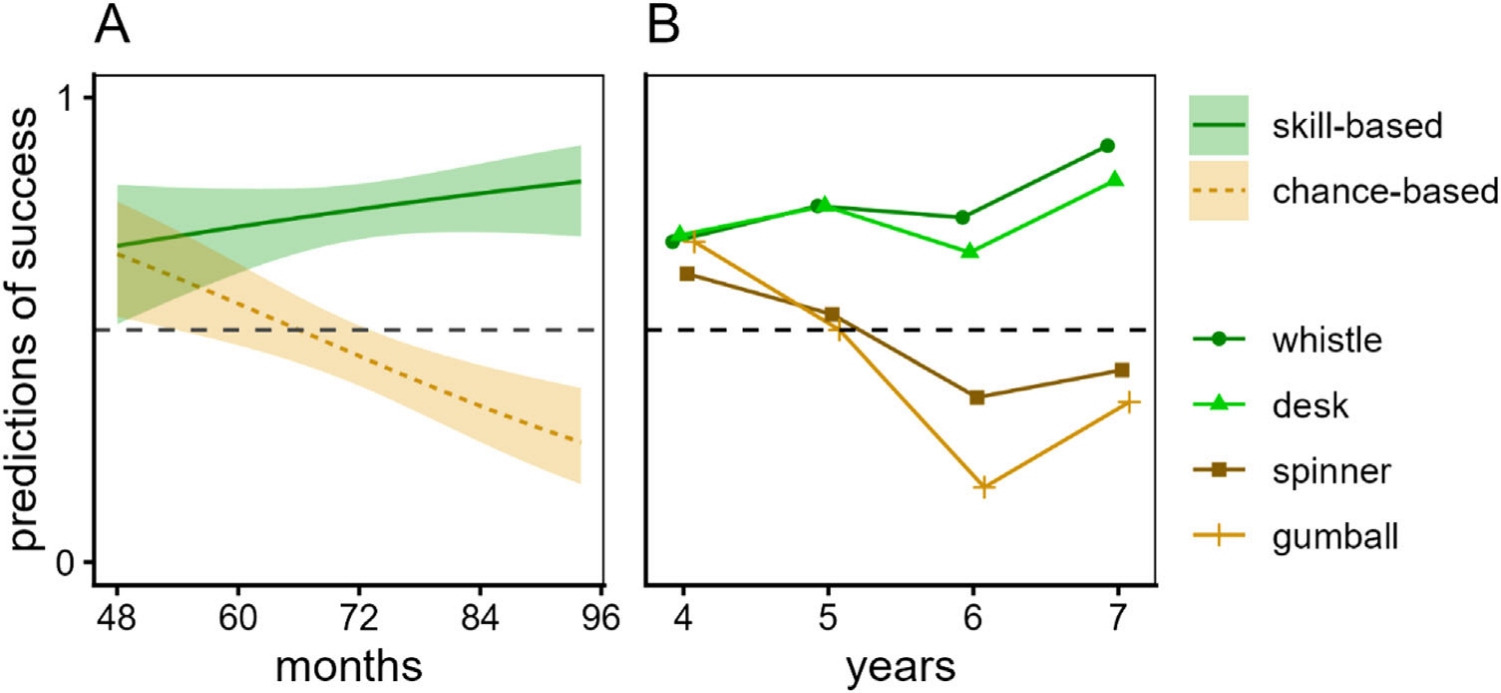
Experiment 1: Children's predictions of success on next attempt. Children predicted whether previously successful agents would succeed on their next attempts of skill‐ and chance‐based activities. (A) Children's predictions of success (with 95% confidence intervals) as predicted by the GEE model. (B) Mean responses across the individual items at each age in years.

We entered predictions of whether the agent's next attempt would succeed (scored as 1) or fail (scored as 0) into a GEE model with activity‐type (skill, chance) and age in months as predictors; see Figure 2 (also see the Supporting Information for means and standard errors binned by age in years). There was a main effect of activity‐type, *F*(1, 468) = 52.10, *p* < 0.001, which resulted because children more often predicted success for skill‐ than chance‐based activities. There was no main effect of age, *F*(1, 468) = 1.10, *p* = 0.290, but the interaction between activity‐type and age was significant, *F*(1, 468) = 15.90, *p* < 0.001: With skill‐based activities age did not significantly affect judgments, *p* = 0.217, whereas with chance‐based ones older children were more likely than younger ones to deny future success, *p* < 0.001.

Examining confidence intervals suggested that responses across the two activity types diverged by Age 5;2 (62 months): skill‐based, 95% CI [0.64, 0.80]; chance‐based, 95% CI [0.45, 0.62]. A single‐sample test (via a no intercept model) showed that children mostly thought the agent's next attempt at a skill‐based activity would succeed, *p *< 0.001; children mostly thought the next attempt at a chance‐based activity would fail at Age 6;2 (74 months, 95% CI [0.36, 0.49]).

### 4.3 Discussion

These findings suggest that starting at Age 5, children think that past success is predictive of future performance in skill‐ but not chance‐based activities. However, in this experiment we only showed children situations in which an agent succeeded, but what about failed attempts? With skill‐based activities the agents’ record of success or failure should matter, since it is indicative of the agents’ competence with the activities. By contrast, with chance‐based activities, whether the agent previously succeeded or failed is largely immaterial, since past and future performance depend on chance alone. In our next experiment, we examine this understanding by testing if children's predictions differ based on whether the agent had recently succeeded or failed.

## 5 Experiment 2

### 5.1 Methods

#### 5.1.1 Participants

We tested 160 4–7‐year‐olds (*M*_age_ = 5;11, range = 4;0–7;11, 80 girls and 80 boys). Three other children were tested but excluded because they did not give “yes” or “no” responses on most test trials.^4^ In this experiment, we aimed to test 20 participants per age in years in each between‐subjects condition, but the actual numbers slightly departed from this. We also tested 202 adults (*M*_age_ = 40 years, range = 18–77; 113 men, 86, women, 3 indicating or preferring not to disclose); a further 10 adults were also tested but excluded for failing comprehension questions.

#### 5.1.2 Materials and Procedure

**FIGURE 3:**
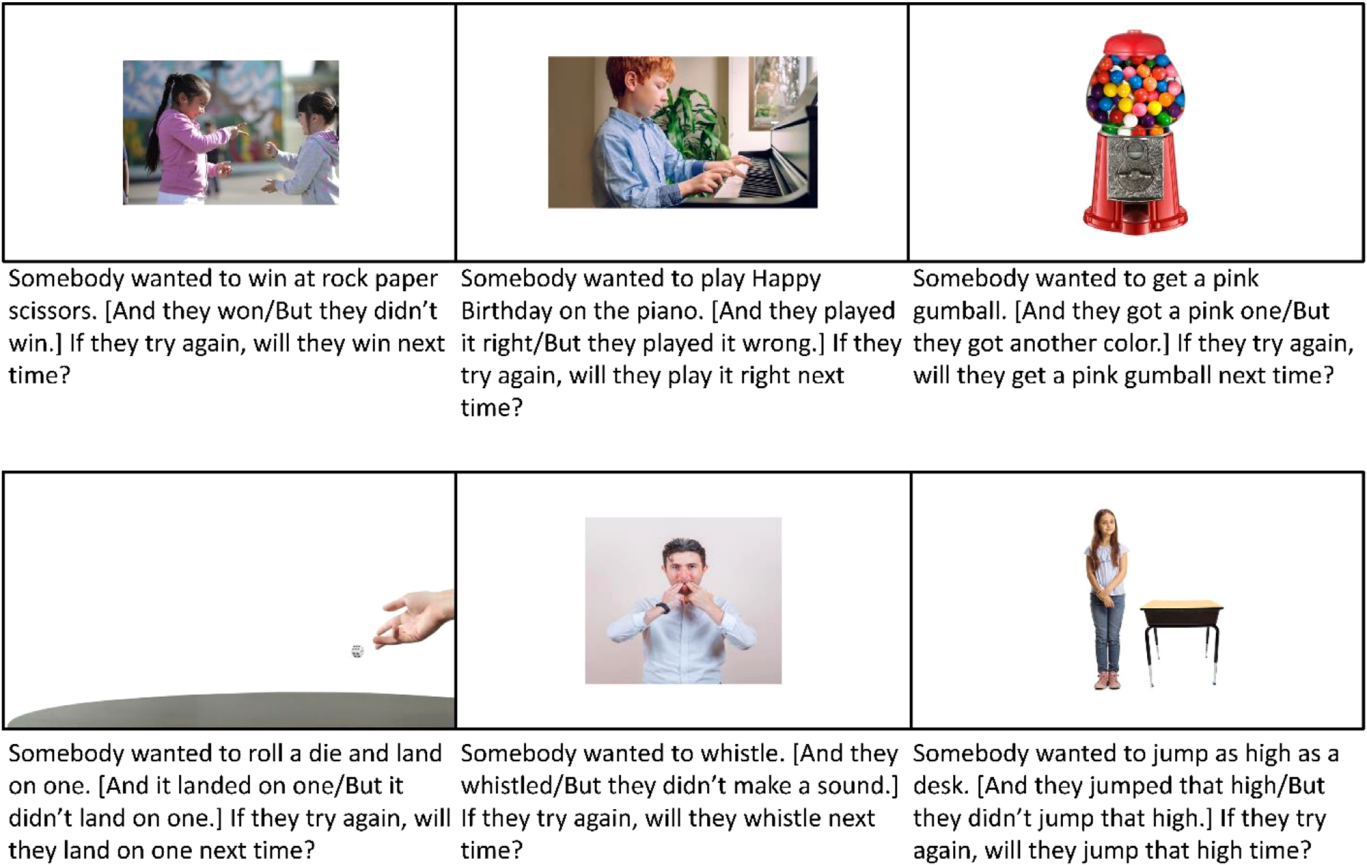
Experiment 2: Slides and testing script. Text in square brackets varied across the two between‐subjects conditions. Children completed the trials in the fixed order shown here (top row, then bottom row) or in the order: piano, rock paper scissors, whistle, jump, gumball, die. For adults, trial order was randomized.

Children completed six trials, with three showing skill‐based activities and the other three showing chance‐based ones; see Figure 3. In each trial, children were either told that someone had succeeded at the activity or had failed (manipulated between‐subjects). They were then asked to predict how the agent would perform if they tried again. As before, the experimenter did not provide feedback on children's responses. Adults completed the same procedure but in a self‐administered survey administered using Qualtrics.

### 5.2 Results

**FIGURE 4:**
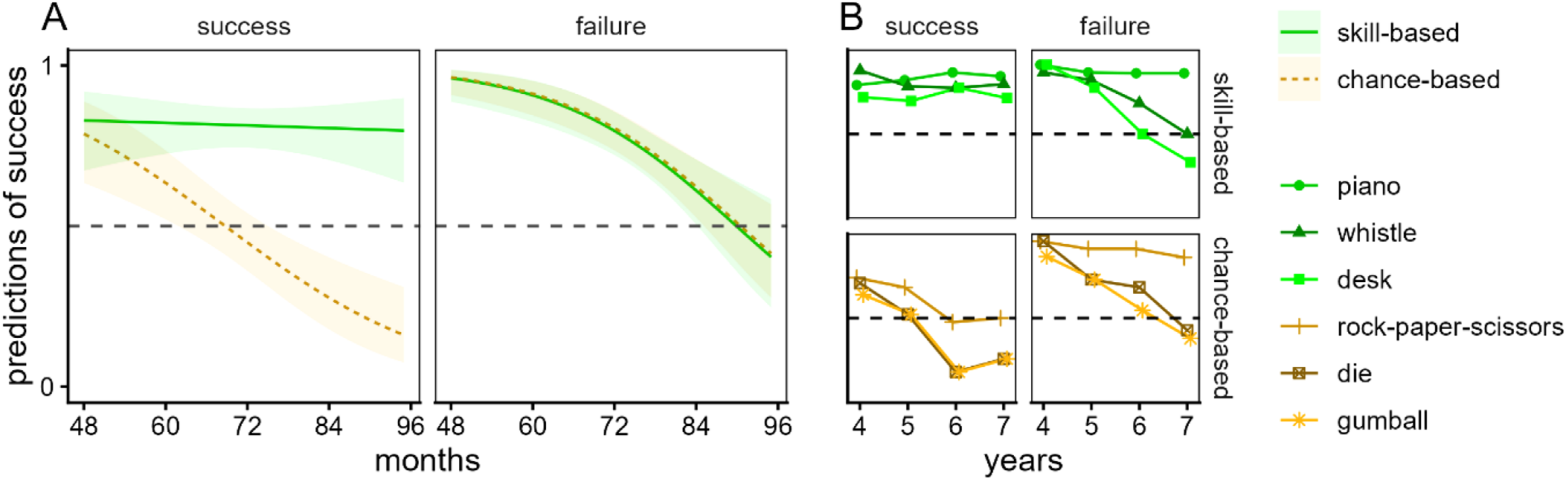
Experiment 2: Children's predictions of success on next attempt. Children predicted whether previously successful or unsuccessful agents would succeed on their next attempts of skill‐ and chance‐based activities. (A) Children's responses (with 95% confidence intervals) as predicted by the GEE model. (B) Mean responses across the individual items at each age in years.

We first examined children's responses. We entered their predictions of whether the agent's next attempt would succeed (scored as 1) or fail (scored as 0) into a GEE model with activity‐type (skill, chance), previous‐attempt (success, failure), and age in months as predictors; see Figure 4 (also see the Supporting Information for means and standard errors binned by age in years). There was a main effect of previous‐attempt, *F*(1, 939) = 12.90, *p* < 0.001, a main effect of activity‐type, *F*(1, 939) = 17.30, *p* < 0.001, a main effect of age, *F*(1, 939) = 38.80, *p* < 0.001, an interaction between previous‐attempt and activity‐type, *F*(1, 939) = 19.80, *p* < 0.001, an interaction between previous‐attempt and age, *F*(1, 939) = 5.80, *p* = 0.016, and an interaction between activity‐type and age, *F*(1, 939) = 3.90, *p* = 0.049; the three‐way interaction fell short of significance, *F*(1, 939) = 3.80, *p* = 0.051.

To make sense of these interactions, we ran separate activity‐type by age analyses for children at each level of previous‐attempt. For children who were asked about agents who had succeeded, there was a main effect of activity‐type, *F*(1, 939) = 39.80, *p* < 0.001, a main effect of age, *F*(1, 939) = 8.40, *p* = 0.004, and a significant interaction, *F*(1, 939) = 7.70, *p* = 0.006. The interaction resulted because there was no effect of age for skill‐based activities, *p* = 0.780, whereas for chance‐based activities predictions of future success decreased with age, *p* < 0.001. For children asked about agents who had failed, there was only a significant main effect of age, *F*(1, 939) = 33.10, *p <* 0.001 resulting because older children were likelier than younger ones to deny future success. There was no main effect of activity type, *F*(1, 939) = 0.00, *p *= 0.846, and no interaction, *F*(1, 939) = 0.00, *p* = 0.990.

We also ran separate previous‐attempt by age analyses for each level of activity‐type. For skill‐based activities, there was no main effect of previous‐attempt, *F*(1, 939) = 0.00, *p* = 0.965, but there was a main effect of age, *F*(1, 939) = 10.90, *p *< 0.001, and a significant interaction, *F*(1, 939) = 8.70, *p* = 0.003, which resulted because there was no effect of age with past success (as reported above), but an age‐related decrease in predictions of future success with failure. For chance‐based activities, there was a main effect of condition, *F*(1, 939) = 34.40, *p *< 0.001, and a main effect of age, *F*(1, 939) = 41.10, *p* < 0.001, but the interaction was not significant, *F*(1, 939) = 0.40, *p* = 0.535. As Figure 4 shows, children more often predicted success after past failure than after past success, though with age children increasingly denied future success.

Examining confidence intervals suggested that when the agent had previously succeeded, responses for the two activity types diverged by Age 5;1 (61 months): skill‐based, 95% CI [0.73, 0.89]; chance‐based, 95% CI [0.51, 0.72]. With skill‐based activities, children overall thought past success would be matched with future success; with chance‐based activities they predicted future success at chance rates by Age 5;2 (62 months, 95% CI [0.50, 0.70]). When agents failed, younger children predicted future success, and predictions only declined to chance rates at Age 7;2 (86 months, 95% CI [0.49, 0.66]).

#### 5.2.1 7‐Year‐Olds Versus Adults

**FIGURE 5:**
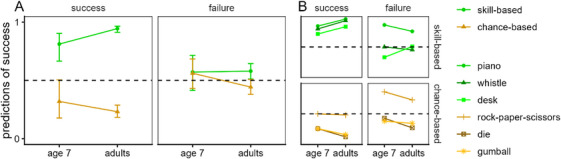
Experiment 2: Older children's and adults’ predictions of success on next attempt. (A) Predictions of success from 7‐year‐olds and adults (with 95% confidence intervals) as predicted by the GEE model comparing their responses. (B) Mean responses across the individual items for each age group.

To get a sense of development across the lifespan, we compared 7‐year‐olds’ predictions of future success with those of adults using a GEE model with the predictors activity‐type (skill, chance), previous‐attempt (success, failure), and age category (7‐year‐olds, adults); see Figure 5. There was a main effect of activity type, *F*(1, 1415) = 69.40, *p* < 0.001, a main effect of previous‐attempt, *F*(1, 1415) = 5.30, *p* = 0.020, but no main effect of age category, *F*(1, 1415) = 0.40, *p* = 0.508. Two of the 2‐way interactions were significant: activity‐type by age category, *F*(1, 1415) = 8.00, *p* < 0.005, and activity‐type by previous‐attempt, *F*(1, 1415) = 47.50, *p* < 0.001. The interaction between activity‐type and age category was not significant, *F*(1, 1415) = 3.40, *p* = 0.064, while the 3‐way interaction was significant, *F*(1, 1415) = 2.50, *p* = 0.112.

The interaction between activity‐type and age category resulted because in comparison with 6–7‐year‐olds, adults were overall more likely to predict future success for skill‐based activities, OR = 0.50, *p* = 0.016, whereas their responses did not significantly differ for chance‐based activities, OR = 1.58, *p* = 0.077. Also, the interaction between activity‐type and previous‐activity resulted because participants were much more likely to predict success for skill‐ than chance‐based activities following past success, OR = 22.73, *p* < 0.001, whereas their predictions did not differ following failure, OR = 1.34, *p* = 0.137.

### 5.3 Discussion

When children considered prior success, they again differentiated between skill‐ and chance‐based activities starting at Age 5. Children at all ages predicted that prior success with skill‐based activities would persist in the future, while older children increasingly denied that prior success with chance‐based activities would persist. Things were different when children considered prior failure. Here, they responded similarly for both activity types, predicting future success at relatively high rates, though this declined with age. Comparing older children's responses with those of adults also suggested that predictions of future success continue to develop past ages 6–7, though overall their responses were quite comparable.

## 6 General Discussion

In two experiments, we examined young children's developing ability to distinguish between activities that depend on skill, and those which involve chance alone. Children predicted how agents who previously succeeded (or failed) with skill‐ and chance‐based activities would perform on their next attempt. In both experiments, children distinguished between skill‐ and chance‐based activities just after they turned 5; both experiments identified Age 5;1 or 5;2 as the average age at which children first recognized past success as having greater predictive utility for skill‐ than chance‐based activities. From this age, children were more likely to predict future success for an agent who had succeeded in jumping as high as a desk than for an agent who had won at rock paper scissors. Children aged 4, by contrast, predicted future success at high rates for both kinds of activities.

Children's differential predictions across activity‐types suggest they recognize that success with some activities is indicative of competence (i.e., skill), whereas success with other activities is not indicative of this. On this view, the findings suggest a change in children's theory of performance at Age 5. Even so, performance continued to improve after Age 5. Older children more reliably recognized that past success with luck‐based activities did not predict future success, and even 7‐year‐olds’ responses differed somewhat from those of adults (i.e., for previously successful agents). Some of this development could reflect individual differences in when children first distinguish between the two activity types; some children may only first distinguish between the skill and chance‐based activities at Age 6 or even 7. But improvement with age could also indicate protracted development in how children distinguish between the activities.^5^

The findings are open to other explanations, however. Rather than reflecting a theory of performance and an understanding of the distinction between skill and chance, children might have based their responses on statistical learning and previously observed regularities. For instance, children may have noticed that people who manage to whistle typically manage to whistle again on their next attempt whereas people who win a round of rock paper scissors do not reliably win the next round. On this view, protracted development after Age 5 might simply result from children gaining more experiences and observations with the kinds of activities we asked about. Older children will have more experience with activities like whistling and rolling dice than younger children, and on any account our tasks probably would not have been viable if children had been unfamiliar with the activities.

Some findings from the second experiment may speak against this statistical learning account. This experiment examined predictions for previously successful and unsuccessful agents. For both skill‐ and chance‐based activities, younger children were more likely to predict future success when agents previously failed than succeeded. These predictions are unlikely to correspond with observed regularities, though admittedly the findings do not provide positive evidence of children consulting a theory of performance. Future work might test between the accounts by asking children to explain their responses.^6^ Children's explanations have long been seen as indicative of their intuitive theories (e.g., Gopnik and Wellman 1994; Noles and Gelman 2011). Support for the view that children have a theory of performance would be provided if children refer to factors like skill and luck when explaining success and failure with different kinds of activities.

Returning to the findings of the second experiment, why did younger children predict that previous failure would be followed by success? One explanation is that younger children optimistically hoped that a person would not fail twice in a row. Broadly consistent with this, an earlier study yielded similar results: After learning about agents who had previously failed (or succeeded) several times, children often predicted the agents would succeed in future attempts (Boseovski et al. 2009). Older children responded differently. They recognized that previously unsuccessful agents would not be especially likely to succeed, and 7‐year‐olds’ responses for previously unsuccessful agents did not significantly differ from those of adults. It is possible, though, that for both children and adults alike, predictions for previously unsuccessful agents were subject to the gamblers’ fallacy or some similar bias. This is suggested in part by visual inspection of the item‐level responses. For instance, both 7‐year‐olds and adults were more likely to predict future success with rock paper scissors for previously unsuccessful than previously successful agents.

The present findings open several directions for future research. We examined children's recognition of the predictive utility of past success with skill‐ and chance‐based activities. But there are many *other* ways that children might distinguish between these two types of activities. For instance, children might distinguish between them in terms of expertise. Much as people have expertise in different areas of knowledge (e.g., Danovitch and Keil 2004; Keil et al. 2008), they also differ in their skills, and children show some understanding of this in their judgments about help—they understand that people vary in their need for help and in their capacities as helpers (e.g., Baer and Odic 2021; Kushnir et al. 2013; Magid et al. 2018). But to the extent that rolling dice and flicking spinners are chance‐based, they are not open to expertise, and so future work could investigate whether children distinguish between skill‐ and chance‐based activities in this way. Future work could likewise test if children might recognize that practice increases the likelihood of future success with skill‐ but not chance‐based activities. Children might plausibly recognize this difference as even 4‐year‐olds spontaneously mention that practice sometimes matters for success (Cimpian and Markman 2011). Looking at various ways that children come to distinguish between skill‐ and chance‐based activities will be helpful for determining the nature of the developmental improvements we observed. If different measures all converge on the conclusion that children first distinguish between skill and chance at Age 5, this could suggest a conceptual shift in their theory of performance at this age (Carey 2009). Alternatively, if different measures find success at varied ages, this would suggest that understanding of the distinction develops in a piecemeal fashion.

Besides looking at competence, research could explore children's understanding of other dimensions on which tasks differ. For instance, future work could investigate children's recognition of how much effort tasks require. Many studies have looked at whether children can recognize that agents differ in effort and ability, with earlier work suggesting that younger children conflate effort and ability (Folmer et al. 2008; Fry and Duda 1997; Heyman et al. 2003; Heyman and Compton 2006; Nicholls 1978; for a review see Cimpian 2017). More recent work, though, finds that young children can distinguish effort from ability—children aged 4–6 sometimes recognize that an agent who succeeds with little effort is more skilled than one expending more effort to succeed (Muradoglu and Cimpian 2020). So much as we found that children distinguish between tasks involving skill and chance, they might also distinguish between tasks requiring much or little effort. Again, the timing of children's success with this could be informative about convergent development across different judgments.

Future research could also ask children for more fine‐grained predictions. Rather than asking children to predict the outcome of the agent's next attempt, they might be asked to assess the likelihood of success. Examining this could help clarify, for instance, whether participants recognized that different chance‐based activities offer different odds of success. Winning at rock paper scissors is, for instance, more likely than rolling a die to land on one. The item plots from the second experiment suggest that children aged 5 and older recognize that the rock paper scissors offered better odds of success than the other chance‐based activities, but a more systematic exploration would be needed to confirm this.

## 7 Conclusion

In sum, the findings of two experiments suggest that from Age 5, children understand that past success is more predictive of future success for skill‐ than chance‐based activities. The findings suggest an advance in children's theory of performance wherein children move from thinking similarly about chance‐ and skill‐based activities to increasingly recognizing that success in chance‐based activities is not affected by agents’ abilities.

## Funding

This research was supported by separate grants from the Natural Sciences and Engineering Research Council of Canada awarded to O.F. and S.D.

## Ethics Statement

This research was approved by the Office of Research Ethics at the University of Waterloo (Project 30395: Social Understanding in Children).

## Conflicts of Interest

The authors declare no conflicts of interest.

## Supporting information




**Supporting file 1**: desc70123‐sup‐0001‐SuppMat.docx.

## Data Availability

Data and code are at https://osf.io/kup2b/. These experiments were not preregistered.

## Endnotes

1 As will also be reviewed, a few studies did directly compare children's understanding of the two kinds of situations by contrasting knowledgeable choices and pure guesses. While the comparison of informed choices and uninformed guesses is one instantiation of this kind of distinction, the contrast between skill versus chance goes beyond this.

2 From Age 10 months, infants also show this understanding (e.g., Denison and Xu 2014; Téglás et al. 2007; Xu and Garcia 2008). We focus here on reviewing findings with young children since success in infancy is not always matched by success in preschoolers (e.g., Girotto et al. 2016).

3 In other work, we attempted a different but problematic approach. We showed children pairs of skill‐ and chance‐based activities and asked them about which required more effort. This approach was ill conceived, though, since some skill‐based activities require little effort (e.g., they are not physically or mentally demanding) while some chance‐based activities require much.

4 Of these three excluded children, two said “maybe” on most trials. Also, five children included in the main analysis said “maybe” once or twice, so their responses for these trials could not be included in the analysis.

5 Somewhat similarly, while many researchers have seen children's coming to attribute false beliefs at around Age 4 as indicative of a conceptual change in their theory of mind (e.g., Wellman et al. 2001), theory of mind continues to develop (e.g., Lagattuta et al. 2015).

6 Children were often asked to provide explanations in earlier work on skill‐ and chance‐based activities (e.g., Weisz et al. 1982), though the earlier studies did not reveal success as early as found here.
