# Supplementary material for: The Predictive Utility of Past Success: Skill and Chance in Children's Theory of Performance
Source: Dev Sci. 2025 Dec 31;29(2):e70123. doi: 10.1111/desc.70123 (PMC12754640; doi:10.1111/desc.70123)
Supplement: Supplementary file 1 — Supporting file 1: desc70123‐sup‐0001‐SuppMat.docx. [file DESC-29-e70123-s001.docx]

**Supplementary Materials**

***The predictive utility of past success: Skill and chance in children’s theory of performance***

**Table S1**

*Experiment 1: Means and Standard Errors with Age Binned by Years*

| condition | age | mean | SE |
| --- | --- | --- | --- |
| skill-based | 4 | 0.70 | 0.06 |
|  | 5 | 0.77 | 0.06 |
|  | 6 | 0.71 | 0.06 |
|  | 7 | 0.86 | 0.05 |
| chance-based | 4 | 0.66 | 0.06 |
|  | 5 | 0.52 | 0.07 |
|  | 6 | 0.26 | 0.06 |
|  | 7 | 0.38 | 0.06 |

**Table S2**

| outcome | activity | age | mean | SE |
| --- | --- | --- | --- | --- |
| success | skill-based | 4 | 0.82 | 0.05 |
|  |  | 5 | 0.79 | 0.05 |
|  |  | 6 | 0.83 | 0.05 |
|  |  | 7 | 0.81 | 0.06 |
|  | chance-based | 4 | 0.71 | 0.06 |
|  |  | 5 | 0.58 | 0.06 |
|  |  | 6 | 0.25 | 0.06 |
|  |  | 7 | 0.32 | 0.07 |
| failure | skill-based | 4 | 0.93 | 0.03 |
|  |  | 5 | 0.85 | 0.05 |
|  |  | 6 | 0.70 | 0.06 |
|  |  | 7 | 0.57 | 0.07 |
|  | chance-based | 4 | 0.97 | 0.02 |
|  |  | 5 | 0.82 | 0.05 |
|  |  | 6 | 0.73 | 0.06 |
|  |  | 7 | 0.56 | 0.07 |
